# Workplace bullying and violence in health sector in Saudi Arabia

**DOI:** 10.1097/MD.0000000000034913

**Published:** 2023-09-01

**Authors:** Aseel Khaled Alhassan, Reem Tarik AlSaqat, Fahad Saleh AlSweleh

**Affiliations:** a Dental Department, King Khalid Hospital in AlKharj Ministry of Health, Riyadh, Kingdom of Saudi Arabia; b Dental Clinic, Restorative Division, Princes Noura University, Riyadh, Kingdom of Saudi Arabia; c Dental University Hospital, King Saud University, Riyadh, Kingdom of Saudi Arabia.

**Keywords:** abuse, bullying violence, healthcare workers, KSA, survivors

## Abstract

Workplace bullying violence (BV), with psychological and physical impacts, is increasing globally. However, studies from Saudi Arabia investigating which specialties are most exposed, and linking them with other factors, such as sociodemographic conditions, are scarce. This study aimed to estimate the prevalence of workplace BV over a 12-month period and determine the circumstances related to the event, consequences for the attacker, and targeted personnel among all healthcare providers in the Kingdom of Saudi Arabia (KSA). This analytical cross-sectional study included all health providers registered with the Saudi Commission for Health Specialties who worked for more than 1 year in the healthcare sector (governmental or private) in the KSA until May 2019. In total, 7398 healthcare workers were electively enrolled in the study; 51.3% were men, and 48.7% were women, with a mean age of 40 ± 8.62 years. They were mostly (60%) non-Saudi. Overall, 26.6% encountered BV. Those who worked in the private sector, in shifts, especially evening shifts, were significantly more exposed. Furthermore, pharmacists had the highest prevalence of workplace violence. The prevalence of BV is moderately high; however, it is a serious issue faced by healthcare workers, especially those working night shifts. Pharmacists were more likely to experience bullying. This demonstrates that more support, specific strategies, and policies are required to reduce the occurrence of workplace BV, protect healthcare providers, and prevent attacks. Underreporting these situations may give an incorrect indication of the magnitude of the problem; thus, more education and further studies in the KSA are needed.

## 1. Introduction

In the last decades, a prominent shift in the prevalence of workplace violence (WPV) has been observed globally.^[1–3]^ According to the World Health Organization, violence is defined as “the intentional use of physical force or power, threatened or actual, against oneself, another person, or a group or community, that either result in or has a high likelihood of resulting in injury, death, psychological harm, maldevelopment, or deprivation.”^[1]^ WPV is divided into 2 major groups: physical and psychological violence, which includes verbal, sexual, racial harassment, and bullying or mobbing.^[1]^ Among other types, bullying is defined as “repeated and over time offensive behavior through vindictive, cruel, or malicious attempts to humiliate or undermine an individual or group of employees.”^[2]^ Internationally, physical and verbal violence is gaining more attention and focus, and bullying is a significant problem faced by healthcare workers (HCWs).^[3]^ Globally, the definition of violence varies because of cultural diversity between countries.^[2]^ This could affect the event recognition and, subsequently, its real magnitude. Bullying can be expressed in overt and covert ways. Overt is by directly humiliating the victim, criticizing, or offending them, while covert is indirect behavior of telling rumors, unfair treatment, or ruining their work.^[4]^ In the Arabian Gulf area, specifically Saudi Arabia and the United Arab Emirates, 37.6% of emergency department (ED) workers were exposed to bullying,^[5]^ while only 3.1% of nurses working in psychiatric hospitals in Taiwan were victims of bullying.^[6]^ A quantitative review conducted to estimate and compare different world regions regarding exposure to bullying found it to be 39.7%, with most of the incidences in the Middle East.^[7]^ Another study in Portugal found that only 8% of HCWs, in general, experienced bullying, with the highest cases reported among nurses.^[8]^ Furthermore, 65.84% of Poland nurses with at least 6 months of work experience had experienced bullying.^[9]^ Internationally, women are more exposed to bullying compared to men.^[6,8–10]^ A study conducted in Spain found that HCWs who work in shifts, who are not interested in their job duties, are stressed, unhappy with the work environment, and those whose efforts are not appreciated are more exposed to workplace bullying.^[11]^ A review on the bullying of nurses showed that colleagues in a senior position would bully their peers more,^[3]^ similar to a study conducted among ED staff, where most of the bullying originated from the management followed by a staff member^[12]^

Exposure to workplace bullying has negative impacts on patient safety, like ignoring patients or having ineffective communication, leading to improper medications and/or treatment^[13]^ It can also cause low job performance, insomnia,^[14]^ sleep disturbance,^[15]^ impaired psychological health conditions,^[16]^ anxiety, melancholy, and some physical symptoms.^[17]^ Additionally, a systemic review showed that workers exposed to bullying are more likely to take sick leave.^[18]^ This will cost the workplace more expenditures to cover the shortage, under-productivity, and legal expenses, if necessary,^[4]^ besides the cost of rehabilitating the victim to overcome the side effects of the incident.^[2]^ Moreover, bullying increases the intention to quit jobs.^[14]^

Previous studies have focused on exploring workplace bullying in environments and specialties with a high risk in the main cities of Saudi Arabia. However, the results cannot be generalized to the whole country. In addition, no previous study has covered all specialties.

Furthermore, there is a scarcity of evidence regarding the association between WPV and independent risk factors such as sociodemographic information, working conditions, and hospital violence report guideline characteristics.

Therefore, this study aimed to determine the prevalence of workplace bullying over a period of 12 months, the circumstances related to the event, the consequences for the attackers, and the target persons at all healthcare provider facilities in Saudi Arabia. Moreover, we aimed to identify which group of HCWs was most susceptible.

## 2. Methods

### 2.1. Data collection

This study is part of a research project focused on evaluating WPV in the health sector in Saudi Arabia.^[19]^ It included all healthcare providers who were registered with the Saudi Commission for Health Specialties (SCFHS) and had been working for more than 1 year in the health sector (governmental or private) in Saudi Arabia by May 2019. The exclusion criteria were students, interns, employees of the administrative department, and providers who were not registered with SCFHS or had <1 year of work experience. A convenient sampling technique was used where all eligible participants (i.e., physicians, pharmacists, nurses, midwives and health specialists, healthcare technicians, and technicians) were invited to participate in the study. Overall, 304,002 healthcare providers met the eligibility criteria.

The data were collected using a modified self-administered questionnaire developed by the Joint Program on WPV in the Health Sectors of the World Health Organization, International Labor Organization, International Council of Nurses, and Public Services International. The questionnaire was translated into Arabic for staff who were not fluent in English. Questions that did not apply to Saudi Arabia were omitted.

A pilot test was conducted by distributing the questionnaire to 5 physicians, 5 dentists, 5 nurses, and 5 pharmacists, who were Arabic and English speakers and had the clinical experience to validate the Arabic translation to avoid any misunderstandings; these practitioners were excluded from the main study.

The questionnaire included questions related to the respondents demographic data, workplace characteristics, the experience of violent events during the previous 12 months, risk factors contributing to WPV, personal opinions, perceptions, attitudes, experiences, and participants knowledge of WPV. The researchers distributed the questionnaire through email to the study population. To increase the response rate, the researchers sent reminder emails to the participants after 2 weeks.

### 2.2. Statistical analysis

Data analysis was performed using the SPSS version 22 (IBM, Armonk, NY). Descriptive statistics (frequency and table) were used to describe the basic features of the data. Continuous variables were expressed as mean and standard deviation, whereas categorical variables were expressed as frequencies and percentages. The Kolmogorov–Smirnov statistical test of normality and histograms were used to assess the statistical normality assumption of metric variables. The statistical homogeneity of variance assumption was evaluated using Levene test of homogeneity of variance. The chi-square test of independence was used to explore the correlations between the categorical variables. An independent samples *t* test was used to assess the mean differences of continuous variables across the levels of categorically binary measured variables.

A multivariate binary logistic regression analysis was conducted to assess the combined and individual associations between the relevant predictors of the exposure of HCWs to recent physical violence at the workplace. The association between the measured predictor variables and their outcomes was expressed as an odds ratio with a 95% confidence interval. Statistical significance was set at *P* < .05.

### 2.3. Ethical approval

This study was conducted following the guidelines of the Declaration of Helsinki. Approval was obtained from the institutional review board of King Saud University College of Medicine (approval number: E-18-3391) before the study was started. Written informed consent for participation, publication, and confidentiality was obtained from the study participants at the beginning of the survey.

## 3. Results

### 3.1. Demographic characteristics

Overall, 304,002 HCWs were recruited from the SCFHS database, and 7398 responded to the questionnaire, of which 51.3% and 48.7% were men and women, respectively. The participants mean age was 40 ± 8.62 years; and 60% were of non-Saudi origin. Nurses, midwives, and health specialists accounted for 38.1% of the study population, followed by physicians at 30.91%, healthcare technicians and ambulance technicians at 25.54%, and pharmacists at 5.43%. Most participants were employed full-time (89.86%) in the public or governmental sectors (72.47%) (Table 1).

**Table 1:** Healthcare worker sociodemographic and professional characteristics. N = 7398.

	Frequency	Percentage
Gender		
Male	3792	51.3
Female	3606	48.7
Age		
20–29 yr	402	5.4
30–39 yr	3752	50.7
40–49 yr	2143	29
50–59 yr	882	11.9
≥60 yr	219	3
Nationality		
Saudi	2957	40
Non-Saudi	4441	60
Clinical role		
Physicians	2287	40
Pharmacists	402	5.4
Nurses, midwives, and health specialist	2819	38.1
Healthcare technicians and ambulance	1890	25.5
Rank/seniority		
Junior	4605	62.2
Senior	1876	25.4
Consultant	917	12.4
Experience yr		
1–5 yr	851	11.5
6–10 yr	2334	31.5
11–15 yr	1905	25.8
16–20 yr	1025	13.9
≥21 yr	1283	17.3
Working sector		
Semi-governmental organization	380	5.1
Private sector	1656	22.4
Public/governmental sector	5362	72.5
Employment type		
Full-time	7256	98
Part-time	78	1.1
Temporary/casual	64	0.9

### 3.2. Experience of WPV

Approximately 26.6% of the respondents have been exposed to workplace bullying at least once in the last year. Most HCWs (26.3%) were abused by their managers and/or supervisors, 23.1% were bullied by another staff member, and 20.1% were bullied by their patients. Furthermore, among the bullied persons, 31.5% mentioned they had taken no action, and 28% tried to pretend it had never happened to them (Table 2).

**Table 2:** Healthcare worker perceptions and experience of Bullying at workplace violence.

Variable	Total n (%)
Occurrence of bullying in the last 12 mo, n = 7398	
No	5429 (73.4)
Yes	1969 (26.6)
Typical incident of bullying violence in your workplace, n = 1744	
Yes	1525 (87.4)
No	219 (12.6)
The attacked person, n = 1744	
Management/supervisor	458 (26.3)
Staff member	403 (23.1)
Patient/client	350 (20.1)
Relatives of patient/client	334 (19.2)
External colleague/worker	80 (4.6)
Other persons	79 (4.5)
General public	40 (2.3)
Place of incident, n = 1744	
Inside health institution or facility	1684 (96.6)
Other place	29 (1.7)
Outside (on way to work/health visit/home)	19 (1.1)
At patient/client home	12 (0.7)
Response to the incident, n = 1744	
Took no action	549 (31.5)
Tried to pretend it never happened	488 (28)
Told the offending person to stop	459 (26.3)
Reported it to a senior staff member	383 (22)
Told a colleague	349 (20)
Told my friends/family members	228 (13.1)
Sought counselling	93 (5.3)
Transferred to another position elsewhere	85 (4.9)
Completed an incident/accident report form	73 (4.2)
Took another action	64 (3.7)
Pursued prosecution	16 (0.9)
Sought help from the medical association	8 (0.5)
Completed a compensation claim	9 (0.5)
Sought help from the Saudi Commission for healthcare workers	7 (0.4)
Preventability of incident, n = 1744	
Yes	1158 (66.4)
No	586 (33.6)

### 3.3. Consequences of bullying violence (BV)

Unfortunately, only 11.3% of the bullying events were investigated; the remaining were either not investigated, or the persons involved had no idea whether it was intervened. Actions were taken primarily by the supervisors or managers against 87.1% of the offenders; however, 44.8% of these had a verbal warning, and only 1.7% had their medical care discontinued. Regarding the overall satisfaction with the actions taken by the supervisors, the bullied persons were generally not satisfied. The primary reasons for not reporting the incidents were the belief that it was pointless to report the bullying attack and the fear of negative consequences that may arise from reporting the incident (Table 3).

**Table 3:** Consequences of bullying at workplace violence.

Variable	Total n (%)
Bothering of attack, n = 1744	Mean (SD) Likert rating
a- Repeated, disturbing memories, thoughts, or images of the attack	3.36 (1.29)
b- Avoiding thinking about or talking about the attack or avoiding having feelings related to it	3.29 (1.26)
c- Being “super-alert” or watchful and on guard	3.61 (1.28)
d- Feeling like everything you did was an effort	3.62 (1.27)
Investigating the causes of the incident, n = 1744	
No	1361 (78)
Yes	197 (11.3)
Don’t know	186 (10.7)
The person who makes action, n = 171	
Management/employer	149 (87.1)
Police	13 (7.6)
Other	13 (7.6)
Community	6 (3.5)
Saudi Commission for healthcare specialties	4 (2.3)
Medical association	3 (1.8)
The consequences of the attacker, n = 172	
Verbal warning issued	77 (44.8)
None	49 (28.5)
Don’t know	21 (12.2)
Other	11 (6.4)
Reported to police	7 (4.1)
Aggressor prosecuted	4 (2.3)
Medical care discontinued	3 (1.7)
The offer of employer or supervisor, n = 1280	
Opportunity to speak about/report it	385 (80.9)
Counseling	222 (46.6)
Other support	198 (41.6)
Incident handling satisfaction, n = 1676 Mean (SD) Likert rating, 1 = V dissatisfied, 5 = V satisfied	
Very dissatisfied	932 (55.6)
Dissatisfied	261 (15.6)
Neutral	286 (17.1)
Satisfied	99 (5.9)
Very satisfied	98 (5.8)
Reason for not reporting incident, n = 1676	
I thought it was useless	941 (56.1)
I was afraid of negative consequences	646 (38.5)
I did not know who to report to the incident	218 (13)
It was not important	204 (12.2)
Other	116 (6.9)
I felt ashamed	103 (6.1)
I felt guilty	14 (0.8)

### 3.4. Experience of bullying attacks and their sociodemographic and professional factors

There was no statistically significant association between the HCWs gender and their exposure to bullying, suggesting that both genders may have been exposed to bullying nearly equally (*P* = .350), according to the chi-squared test of independence. Conversely, the HCWs age groups significantly affected their exposure to workplace-related bullying; people aged between 30 and 39 years were significantly more predisposed to workplace bullying compared to workers in other age groups (*P* < .001). In addition, Saudi national HCWs were significantly more exposed to bullying (*P* < .001) compared with non-Saudi HCWs. In addition, pharmacists were significantly more predisposed to bullying than other HCWs (*P* < .001). Moreover, senior HCWs were significantly more predisposed to bullying than junior and consultant HCWs (*P* = .015).

Furthermore, the HCWs years of professional experience significantly affected their exposure to bullying in the workplace (*P* < .001); those with 6 and 10 years of experience were significantly more predisposed to bullying than those with different years of experience. Moreover, HCWs employed in the private sector were significantly more exposed to bullying at work than those in governmental and semi-governmental facilities; however, the type of employment of the HCWs did not correlate significantly with their exposure to bullying at work (Table 4).

**Table 4:** Association between healthcare workers experience of bullying at workplace with their sociodemographic and professional factors.

	Bullied in your workplace n (%)		Test statistic	*P* value
	No = 5429	Yes = 1969		
Gender				
Male	2765 (50.9)	1027 (52.2)	χ^2^ (1) = 0.87	.350
Female	2664 (49.1)	942 (47.8)		
Age				
20–29 yr	281 (5.2)	121 (6.1)	χ^2^ (4) = 77.83	<.001
30–39 yr	2621 (48.2)	1131 (57.4)		
40–49 yr	1623 (29.9)	520 (26.4)		
50–59 yr	715 (13.2)	167 (8.5)		
≥60 yr	189 (3.5)	30 (1.5)		
Nationality				
Saudi	2076 (38.2)	881 (44.7)	χ^2^ (1) = 25.50	<.001
Non-Saudi	3353 (61.8)	1088 (55.3)		
Clinical role				
Physicians	1647 (30.3)	640 (32.5)	χ^2^ (3) = 39.57	<.001
Pharmacists	247 (4.5)	155 (7.9)		
Nurses, midwives, and health specialists	2100 (38.7)	719 (36.5)		
Healthcare technicians and ambulance	1435 (26.4)	455 (23.1)		
Rank/seniority				
Junior	3421 (63)	1184 (60.1)	χ^2^ (2) = 8.34	.015
Senior	1329 (24.5)	547 (27.8)		
Consultant	679 (12.5)	238 (12.1)		
Experience yr				
1–5 yr	629 (11.6)	222 (11.3)	χ^2^ (4) = 40.68	<.001
6–10 yr	1623 (29.9)	711 (36.1)		
11–15 yr	1389 (25.6)	516 (26.2)		
16–20 yr	776 (14.3)	249 (12.6)		
>20 yr	1012 (18.6)	271 (13.8)		
Working sector				
Other semi-governmental/private organization	277 (5.1)	103 (5.2)	χ^2^ (3) = 10.14	.017
Private-for profit sector	1166 (21.5)	490 (24.9)		
Public/governmental sector	3986 (73.4)	1376 (69.9)		
Employment type				
Full-time	5325 (98.1)	1931 (98.1)	χ^2^ (2) = 0.513	.774
Part-time	59 (1.1)	19 (1)		
Temporary/casual	45 (0.8)	19 (1)		

### 3.5. Experience of bullying attacks and their working conditions

Table 5 shows that those who work in shifts, especially evening shifts, were significantly more predisposed to workplace bullying (*P* < .001). Furthermore, HCWs working closely with patients and those whose work involve physical interaction with their patients were significantly more predisposed to bullying (*P* = .001). Moreover, HCWs working with both male and female patients were significantly more predisposed to bullying than those working with male or female patients only or those not working directly with patients (*P* < .001).

**Table 5:** Association between healthcare workers experience of bullying at workplace with their working conditions factors.

Variable	Bullied in your workplace (%), n = 7398		Test statistic	*P* value
	No = 5429	Yes = 1969		
Work in shifts				
No	2447 (45.1)	734 (37.3)	χ^2^ (1) = 35.82	<.001
Yes	2982 (54.9)	1235 (62.7)		
Working time between 18:00 (6 pm) and 07:00 (7 am)				
No	2115 (39)	606 (30.8)	χ^2^ (1) = 41.59	<.001
Yes	3314 (61)	1363 (69.2)		
Interacting with patients/clients				
No	583 (10.7)	164 (8.3)	χ^2^ (1) = 9.24	.002
Yes	4846 (89.3)	1805 (91.7)		
Routine direct physical contact (washing, turning, lifting) with patients/clients				
No	2505 (46.1)	880 (44.7)	χ^2^ (2) = 13.35	.001
Yes	2344 (43.2)	925 (47)		
Not applicable	580 (10.7)	164 (8.3)		
Patients/clients you most frequently work with are (tick all appropriate)				
Newborns	954 (17.6)	416 (21.1)	χ^2^ (1) = 12.1	.001
Infants	1147 (21.1)	466 (23.7)	χ^2^ (1) = 5.50	.019
Children	1952 (36)	799 (40.6)	χ^2^ (1) = 13.23	<.001
Adolescents	2479 (45.7)	1068 (54.2)	χ^2^ (1) = 42.61	<.001
Adults	4232 (78)	1607 (81.6)	χ^2^ (1) = 11.66	.001
Elderly	2984 (55)	1242 (63.1)	χ^2^ (1) = 38.84	<.001
Gender of the patients you most frequently work with				
Unspecified/NA	580 (10.7)	164 (8.3)	χ^2^ (3) = 34.51	<.001
Female	434 (8)	139 (7.1)		
Male	523 (9.6)	127 (6.4)		
Male and female	3892 (71.7)	1539 (78.2)		

### 3.6. Influence of patients age group on exposure to bullying

**Figure 1.**
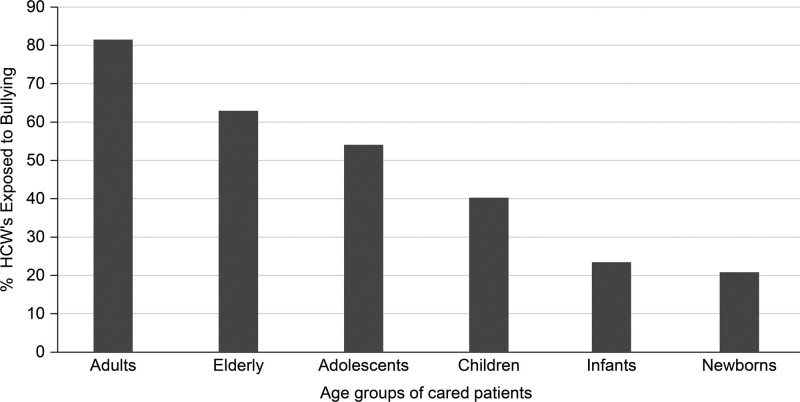
The percentage of healthcare workers exposed to bullying broken down by the age group of the patients they cared for.

Figure 1 shows the percentage of HCWs exposed to bullying by the age group of patients they cared for. All HCWs working with patients in different age groups were significantly more predisposed to bullying; however, the most exposed group of HCWs were those working with adults, followed by those working with older adults, adolescents, children, and infants. The least exposed were those working with newborns.

### 3.7. Experience of bullying attacks and their hospital violence reporting guideline characteristics

HCWs exposed to bullying had significantly greater worries about WPV (mean worry = 3.41 points using a Likert scale, SD = 1.23) than those who had never been bullied (mean worry = 2.64, SD = 1.30; *P* < .001). In addition, HCWs serving at a facility with a guideline for reporting WPV were significantly less predisposed to bullying than those working in places with no policies or guidelines for reporting and/or dealing with intimidating workplace behaviors like bullying (*P* < .001; Table 6). HCWs serving in institutions with guidelines but lacking knowledge of how to use those guidelines for reporting and managing WPV were statistically significantly more prone to bullying than others who knew how to use the guidelines and report the violence (*P* = .003). However, HCWs working in an encouraging environment for reporting violence were significantly less predisposed to bullying than those working in an environment that did not encourage reporting violence (*P* < .001).

**Table 6:** Association between healthcare workers experience of bullying at workplace with their hospital violence reporting guidelines characteristics.

Variable	Bullied in your workplace (%), n = 7398		Test statistic	*P* value
	No = 5429	Yes = 1969		
Worried about violence in the current workplace-Mean (SD)	2.64 (1.30)	3.41 (1.23)	T (7396) = 32.74	<.001
Presence of procedures for reporting of violence				
No	1423 (26.2)	673 (34.2)	χ^2^ (1) = 45.19	<.001
Yes	4006 (73.8)	1296 (65.8)		
knowing how to use report				
No	559 (14)	224 (17.3)	χ^2^ (1) = 8.63	.003
Yes	3447 (68)	1072 (82.7)		
Encouragement to report workplace violence				
No	1724 (31.8)	1015 (51.5)	χ^2^ (1) = 242.80	<.001
Yes	3705 (68.2)	954 (48.5)		
Person who encourages reporting				
Management/employer	3118 (57.4)	814 (41.3)	χ^2^ (1) = 150.25	<.001
Colleagues	1141 (21)	343 (17.4)	χ^2^ (1) = 11.66	.001
Saudi commission for health specialist	444 (8.2)	126 (6.4)	χ^2^ (1) = 6.43	.011
Medical association	147 (2.7)	34 (1.7)	χ^2^ (1) = 5.83	.016
My own family/friends	278 (5.1)	98 (5)	χ^2^ (1) = 0.06	.804
Other persons	230 (4.2)	77 (3.9)	χ^2^ (1) = 0.39	.535

### 3.8. Effect of encouragement sources on exposure to bullying

**Figure 2.**
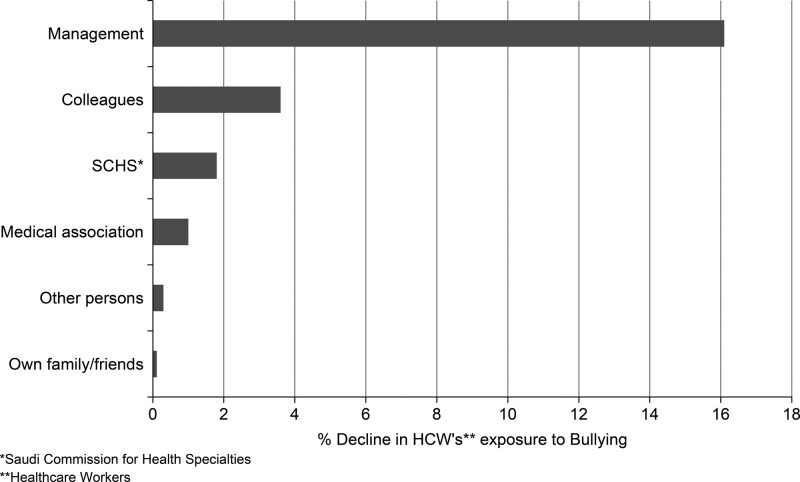
The decline in % in exposure to bullying with encouragement for reporting violence from various sources.

The encouragement from various persons for reporting bullying was statistically significantly correlated with less exposure to bullying (*P* < .001) except for encouragement from family members and others. However, encouragement from management was associated with a substantial decline in the exposure of HCWs to bullying (Fig. 2).

### 3.9. Multivariate logistic binary regression analysis results

**Figure 3.**
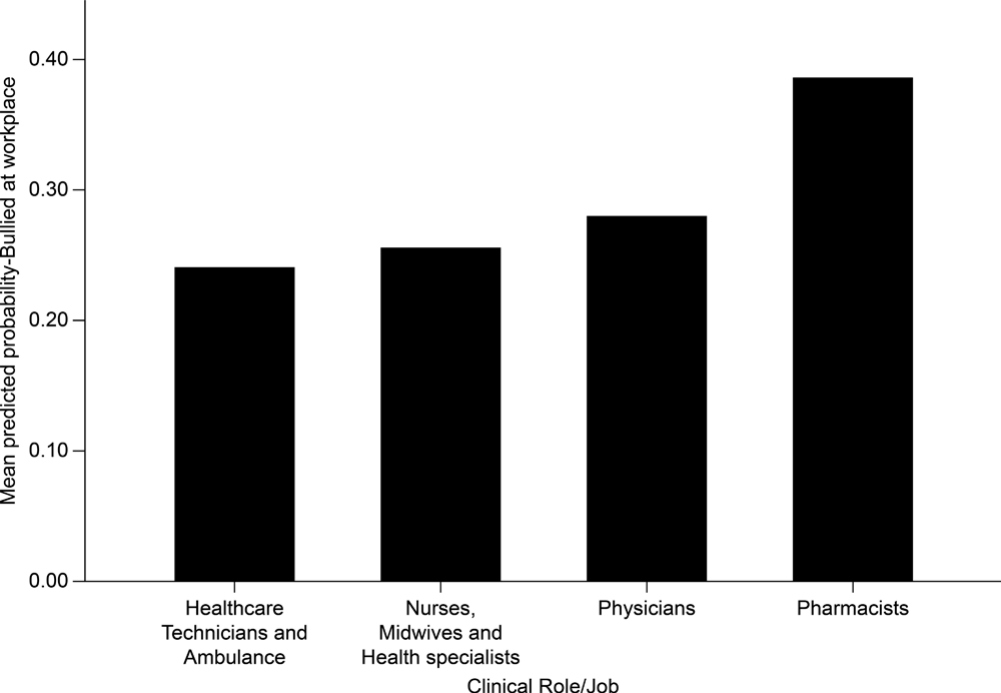
The association between the healthcare workers job with their multivariate adjusted predicted probability of exposure to bullying at workplace.

**Figure 4.**
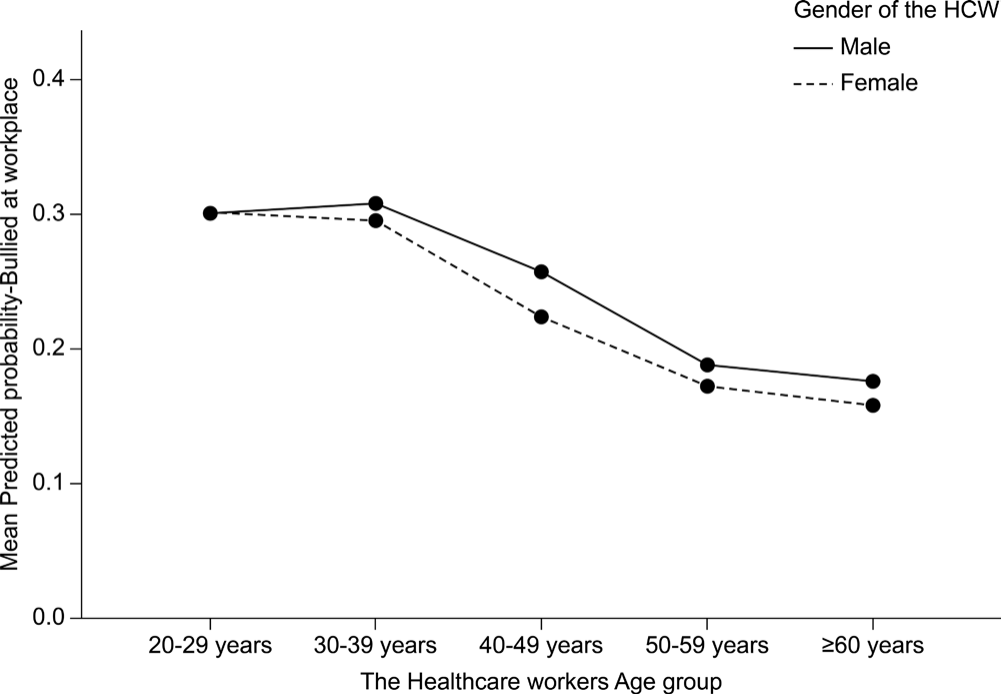
The association between the workers age with their multivariate adjusted predicted probability of exposure to bullying at workplace with the subgroup of the workers gender.

Table 7 shows the multivariate logistic binary regression analysis of the predictors of HCWs exposure to workplace bullying. The HCWs worry level from violence at work mean score had significant positive effects on their odds of exposure to the workplace in the last year. For each 1-unit rise in the HCWs worry score from WPV, their odds of exposure to bullying rose by a factor equal to 46.9% times higher (*P* < .001). Nonetheless, those HCWs who spent >50% of their work time at the hospital were significantly more bullied (46.5% more) compared with those who spent less than half of their work time at hospitals, on average (*P* < .001). Pharmacists were significantly more bullied (44% higher) than physicians (*P* = .004). The nurses were significantly less bullied (20.3% less) than physicians on average (*P* = .004). In addition, technicians and medical technologists had significantly lower odds (26.7% less) of bullying in the last year compared with physicians (*P* < .001) (Fig. 3). Furthermore, the analysis model indicated that HCWs serving in private sector facilities were significantly more predisposed (37.6% more) to bullying in the last year compared with those working in governmental and semi-governmental facilities, on average (*P* < .001). Moreover, female HCWs were significantly more predisposed (16% higher) to workplace bullying than male HCWs, on average (*P* = .019) (Fig. 4). HCWs aged 40 to 49 years were significantly more predisposed (26.7% higher) to bullying than those aged >60 years (*P* = .016). Moreover, the analysis model showed that non-Saudi HCWs were significantly less predisposed to bullying in the workplace (25.4% less) than Saudi HCWs, on average (*P* < .001). HCWs who had direct physical contact with patients at work were significantly more bullied (14.1% more) than those who had no direct physical contact with patients, on average (*P* = .005). The guidelines for reporting and managing WPV for the HCWs did not significantly affect their odds of being bullied in the last year (*P* = .6960). However, the guidelines seem to have some negative but not statistically significant association with HCWs exposure to bullying in the last 12 months. The administrative encouragement to report WPV had significant negative effects on the HCWs exposure to bullying, as those HCWs encouraged by their management and superiors to report violence of any kind were significantly less predisposed (59.9% less) to bullying in the last year compared with those who were not encouraged to report violence by their facility administration (*P* < .001).

**Table 7:** Multivariate logistic binary regression analysis of the predictors of healthcare workers exposure to bullying at workplace, N = 7398.

	Multivariate adjusted odds ratio (OR)	95% C.I. for OR		*P* value
		Lower	Upper	
Encouragement for reporting by significant others	1.569	1.168	2.107	.003
Worry level from violence at work -mean score	1.469	1.406	1.535	<.001
Spends >50% of the work time at hospital	1.465	1.243	1.725	<.001
Job = Pharmacist	1.440	1.125	1.843	.004
Works in a private sector facility	1.376	1.191	1.589	<.001
Aged 30–39 yr	1.359	1.123	1.644	.002
Encouragement for reporting from management/leaders	1.341	1.080	1.664	.008
Aged 40–49 yr	1.267	1.045	1.537	.016
Aged 20–29 yr	1.252	0.932	1.680	.135
Works with newborns	1.233	1.074	1.416	.003
Encouragement for reporting by colleagues	1.186	1.013	1.389	.034
Works with elderly	1.185	1.053	1.333	.005
Works the PM shifts between 18:00 pm and 07:00 am	1.166	1.033	1.317	.013
Gender = Female	1.159	1.025	1.310	.019
Have direct physical contact with the patients at work	1.141	1.040	1.252	.005
Presence of violence reporting guidelines at workplace	0.973	0.850	1.115	.696
Job = Nurse	0.797	0.684	0.930	.004
Nationality = None Saudi	0.746	0.657	0.849	<.001
Job = Technicians and medical technologists	0.733	0.623	0.862	<.001
Administrative encouragement to reporting workplace violence	0.401	0.316	0.509	<.001
Constant	0.105			<.001

## 4. Discussion

WPV is a global epidemic^[20]^ that is also common in Saudi Arabia. Many studies have assessed its prevalence in some Saudi Arabian cities and specialties.^[5,12,21,22]^ However, to our knowledge, no study has focused on HCWs from all cities in Saudi Arabia. This is a comprehensive study with many participants focusing on BV against all HCWs in Saudi Arabia. Our study indicated a significant association between exposure to BV and working as a pharmacist (*P* = .004).

One-quarter of participants in the current study suffered from bullying, which is not approximating other local studies.^[5,12,21]^ El-Gilany et al^[21]^ reported the lowest percentage of bullying but was focused only on primary HCWs in 1 city, which was less equipped with staff and devices; thus, patients seek treatment in the main hospitals in big cities. Conversely, the study by Harthi et al^[12]^ was mainly on ED staff in 1 city with many unreported incidents. In addition, working in such a stressful environment could make HCWs tolerate bullying, as it is considered a part of their jobs. Alshahrani et al^[5]^ reported the highest bullying incidences among all other studies. The study included EDs in Saudi Arabia and the United Arab Emirates, which may differ in system and regulations. Furthermore, most participants had <5 years of experience, which might make them less familiar with dealing with and containing such events. Internationally, the bullying exposure rate ranges from 3.1% to 65.84%.^[6–11]^ This enormous difference could be because different settings and world regions^[7]^ have no clear definition for bullying that could make the victims recognize the incidents^[8]^ and the different methodologies used.^[9]^ Bullying can arise from hostile working environments, fixed routine schedules, demanding working conditions,^[11]^ and having careless coworkers who witness the bullying of their colleagues.^[3]^ In Saudi Arabia, some bullying incidents might be because many non-Arabic speakers are working as HCWs, leading to miscommunication between the native speakers who are unsatisfied with unserviceable communication.^[22]^

Most victims said they were mainly bullied by the management or supervisor, followed by a staff member. Our findings correspond with Harthi et al^[12]^ and Benardes et al^[23]^ though most of the victims in their studies were female nurses. Nurses are more prone to bullying because they are considered to be in a lower position compared with other staff members and are scarcely trained on how to handle such situations.^[4]^ In addition, the fear of being bullied by a higher authority could make them silent on the incident to avoid losing their jobs or having their salary reduced. Other studies reported that most aggressors are patients,^[6,24]^ which could be because of prioritizing patients over workers without sanctioning the attackers.^[12]^ Most victims in the current study took no action against the attackers or pretended that the incident never happened, which corresponds with a study in Brazil.^[23]^ However, most respondents knew how to report such events and were encouraged to. This attitude could be explained by the victims dissatisfaction with the way such incidents are handled, as more than half of the participants thought it was useless to report or were afraid of penalties. The fact that most of those who should be responsible for preventing or stopping these behaviors are the ones most likely to be the perpetrators resulted in underreporting, as the victims had already received the bullying attack from someone in a higher authority position.^[3]^ Moreover, not classifying behavior as bullying would cause it to be underestimated as a serious problem affecting HCWs.^[3]^ Not reporting BV would encourage its continuity and make it even worse^[23]^ There was no statistically significant association between HCWs and their exposure to bullying between men and women. However, when adjusted for sociodemographic, professional, and working conditions, female HCWs were significantly more predisposed to bullying at work than male HCWs (Odds ratio, 1.16, *P* value = 0.019). Most studies have reported that women are more exposed to bullying^[8,10,11,23]^ Some of those studies were mainly about nurses, a female-dominated profession; thus, they had more female participants. Cheung et al^[25]^ found that male nurses were more exposed owing to the increased sensitivity of male nurses, which makes them misinterpret other comments as violence. This study found that Saudi nationals were more exposed to BV compared to non-Saudi nationals, in correspondence with other local studies^[12]^ Feeling more comfortable with the HCWs who speak the patient language and mostly from the same provenance made it easier to express their disappointment which eventually ended as bullying. Pharmacists were significantly more predisposed to bullying than other HCWs (*P* < .001) according to the chi-squared test of association. This finding is not comparable with any local study, as none included all HCW specialties. In Portugal, Norton et al^[8]^ found that nurses were more exposed to bullying than the other 6 HCW groups. This was because nurses were usually the highest in number in all healthcare institutes. They are the first-line HCWs to face patients and for a longer period of time, compared with other professions,^[26]^ making them more prone to BV. Senior HCWs were significantly more predisposed to bullying compared with junior and consultant HCWs. This result is the opposite of a study in Poland,^[9]^ where the least senior nurses experienced more bullying. HCWs with intermediate experience can recognize the bullying attack when it happens; however, they lack the consultant experience in dealing with such a situation. HCWs employed in private sectors were significantly more exposed to workplace bullying than those in governmental and semi-governmental facilities. However, the type of employment did not correlate significantly with their exposure to bullying at work. This finding contradicts that of Macau et al,^[27]^ in which their citizens preferred the more affordable governmental hospitals with an increased patient-to-healthcare worker ratio, waiting time, and unmet needs, which results in BV. In Saudi Arabia, treatment and medications are free; thus, more patients prefer governmental hospital treatment. Therefore, private hospitals give priority to their patients to maintain patient flow. They also tolerate their bad behavior and BV. Bullying attacks were more common among HCWs who work in shifts in correspondence with other studies.^[8,11]^ According to Cheung et al,^[27]^ patients or their relatives tend to bully HCWs because of unmet needs when there is a delay while changing shifts. In addition, having the same HCW in different shifts could make the patients blame them for any unmet need, even if they were not the cause. Conversely, HCWs working night shifts were significantly more predisposed to workplace bullying. This contradicts the findings of Harthi et al,^[12]^ who attributed the bullying in his study to lack of consequences for the offenders.

BV has great consequences on HCWs. It can cause stress, depression, and psychosomatic symptoms. It also affects their daily work by reducing work efficiency, and some may quit their jobs.^[3]^

Social media is an essential factor nowadays, as anonymous harmful comments could easily reach the victims^[23]^ More intensive awareness campaigns about the consequences of bullying should be implemented, periodic checkups of HCWs should be done, and strict preventive policies should be implemented. Further longitudinal studies may be needed to explore the causes of BV and to implement solutions accordingly. Educational programs are required for HCWs, especially those working under supervision, patients, and their relatives. In addition, increasing social media awareness is essential. Environments that encourage the reporting of violent incidents with strict consequences for attackers should be provided. More importantly, new regulations regarding increased staffing, shorter waiting times, and increased support and prevention programs are vital.

The limitations of this study included its retrospective design and use of self-reported questionnaires, which may promote recall bias. Moreover, although this study had a sufficiently large number of participants to be considered a convenience sample, the results cannot be generalized to the general population. On the contrary, the strength of this study was the involvement of all HCWs in both government and private institutions in Saudi Arabia, unlike previous studies that focused on EDs or nurses.

## 5. Conclusions

In our study, we found an average prevalence of bullying WPV. It is a risk faced by HCWs, particularly those working in private sectors, at night, and in shifts. Pharmacists had the highest prevalence of bullying WPV. This study can be considered a basis for future longitudinal studies to implement more support, strategies, and policies to prevent and reduce WPV and protect healthcare providers. By recognizing the most affected groups and those at higher risk, a more supportive environment and guidelines for protection should be implemented. Furthermore, educational programs for HCWs, patients, and their relatives, are required. Under-reporting of WPV incidents may give an incorrect estimation of the magnitude of the problem; therefore, additional education and studies in Saudi Arabia may be needed.

## Acknowledgments

Special thanks to the Saudi Commission for Health Specialties for helping us contact the healthcare providers registered with their institution.

## Author contributions

**Conceptualization:** Fahad Saleh AlSweleh.

**Data curation:** Aseel Khaled Alhassan, Reem Tarik AlSaqat, Fahad Saleh AlSweleh.

**Formal analysis:** Aseel Khaled Alhassan, Reem Tarik AlSaqat, Fahad Saleh AlSweleh.

**Investigation:** Aseel Khaled Alhassan, Reem Tarik AlSaqat.

**Methodology:** Aseel Khaled Alhassan, Reem Tarik AlSaqat, Fahad Saleh AlSweleh.

**Project administration:** Aseel Khaled Alhassan, Fahad Saleh AlSweleh.

**Resources:** Aseel Khaled Alhassan, Reem Tarik AlSaqat, Fahad Saleh AlSweleh.

**Software:** Aseel Khaled Alhassan, Reem Tarik AlSaqat, Fahad Saleh AlSweleh.

**Supervision:** Aseel Khaled Alhassan, Fahad Saleh AlSweleh.

**Validation:** Aseel Khaled Alhassan, Reem Tarik AlSaqat, Fahad Saleh AlSweleh.

**Visualization:** Aseel Khaled Alhassan, Fahad Saleh AlSweleh.

**Writing – original draft:** Aseel Khaled Alhassan, Reem Tarik AlSaqat, Fahad Saleh AlSweleh.

**Writing – review & editing:** Aseel Khaled Alhassan, Reem Tarik AlSaqat, Fahad Saleh AlSweleh.
